# Upregulation of Platelet-Activating Factor Receptor Expression and Lyso-Platelet-Activating Factor Isoforms in Human Nasal Polyp Tissues

**DOI:** 10.3390/jcm12237357

**Published:** 2023-11-28

**Authors:** Jordi Roca-Ferrer, Maria Pérez-González, Isam Alobid, Valeria Tubita, Mireya Fuentes, Marina Bantulà, Rosa Muñoz-Cano, Antonio Valero, Iñaki Izquierdo, Joaquim Mullol

**Affiliations:** 1 Clinical and Experimental Respiratory Immunoallergy (IRCE), Clinic Foundation for Biomedical Research-August Pi Sunyer Biomedical Research Institute (FRCB-IDIBAPS), 08036 Barcelona, Spain; perez5@clinic.cat (M.P.-G.); ialobid@clinic.cat (I.A.); v.tubita@ub.edu (V.T.); mfuentes@recerca.clinic.cat (M.F.); bantula@recerca.clinic.cat (M.B.); rmunoz@clinic.cat (R.M.-C.); valero@somclinic.cat (A.V.); 2 CIBER of Respiratory Diseases (CIBERES), Health Institute Carlos III, 28029 Madrid, Spain; 3 Rhinology Unit & Smell Clinic, ENT Department, Hospital Clínic Barcelona, 08036 Barcelona, Spain; 4 Faculty of Medicine, Universitat de Barcelona, 08036 Barcelona, Spain; 5 Allergy Department, Hospital Clínic, Universitat de Barcelona, 08036 Barcelona, Spain; 6 Clinical Development & Medical Advise, R&D, NOUCOR, 08184 Palau Solità i Plegamans, Spain; inaki.izquierdo@noucor.com

**Keywords:** platelet-activating factor, platelet-activating factor receptor, chronic rhinosinusitis with nasal polyps, asthma, NSAID-exacerbated respiratory disease, Lyso-PAF

## Abstract

Background: The Platelet-Activating Factor (PAF)/receptor (PAFR) system is involved in asthma and allergic rhinitis; however, its role in chronic rhinosinusitis (CRS) is still unclear. This study aimed to assess the expression of PAFR and the concentration of Lyso-PAF isoforms in the nasal polyps (NP) of patients suffering from CRS with/without comorbidities such as asthma and NSAID-exacerbated respiratory disease (N-ERD) compared to healthy nasal mucosa (NM) from control subjects. Methods: NM (*n* = 8) and NP tissues were obtained from patients undergoing surgery for septal deviation/turbinate hypertrophy or endoscopic sinus surgery, respectively. Three phenotypes were studied: CRSwNP with no asthma (*n* = 6), CRSwNP with non-steroidal anti-inflammatory drug (NSAID)-tolerant asthma (*n* = 6), and CRSwNP with NSAID-exacerbated respiratory disease (*n* = 6). PAFR protein and mRNA were assessed via immunochemistry, immunofluorescence, Western blot, and real-time quantitative PCR. Lyso-PAF isoforms (C16, C18, and C18:1) were quantified via mass spectrometry. Results: PAFR protein was expressed in the NM and NP, concretely in epithelial cells and submucosal glands. Compared to NM, PAFR mRNA expression was higher in all NP phenotypes (*p* < 0.05) while all Lyso-PAF isoform concentrations were higher in the NP from asthmatic patients (*p* < 0.05). Lyso-PAF C16 and C18 concentrations were higher in the NP from asthmatic patients than in the NP from patients without asthma. Conclusions: The PAF/PAFR system could play a pathophysiological role in CRSwNP pathogenesis.

## 1. Introduction

Platelet-activating factor (PAF; 1-O-alkyl-2-acetyl-sn-glyceryl-3-phosphocholine) is a phospholipid compound that is normally present in picogram per milliliter concentrations in human serum. PAF plays a relevant role in anaphylaxis, chronic urticaria, and airway inflammatory diseases such as rhinitis and asthma [1].

There are several PAF molecular isoforms, although the most common chemical isoform of PAF in humans is PAF C-16 [2,3]. PAF is synthesized by multiple cells of the immune system, especially eosinophils, neutrophils, and mast cells. However, PAF is not only produced in inflammatory cells but also by structural cells, such as cardiomyocytes, vascular endothelial cells, urothelial cells, and airway epithelial cells [1,3,4]. In fact, upon the PAF being generated, it can act upon the cell itself or to neighboring cells in a juxtracrine fashion [3]. In addition, in some cell types, such as monocytes, neutrophils, and keratinocytes, it has been demonstrated that released PAF exert effects away from the host cell [3]. The exact mechanisms by which cells release PAF are not entirely known, despite studies performed with a keratinocyte cell line that have demonstrated that subcellular microvesicle particles released from the plasma membrane contain PAFR agonistic activity [3]. In fact, traveling in a microvesicle particle may afford protection from degradation by enzymes in comparison to being free or protein-bound in tissue fluids [3].

PAF effects are mediated by the binding to its specific receptor (PAFR). PAF–PAFR engagement activates downstream signaling pathways, including the activation of phospholipases (PLC and PLA2), kinases (such as protein tyrosine kinase and protein kinase C), as well as the production of cytokines, such as TNF-α, IL-1α, and prostaglandins [1]. The differential expression of PAFR in tissues could have pathophysiological relevance. However, there is limited information on PAFR expression in the human upper airways. It has been reported PAFR mRNA and protein expression in nasal mucosa tissue from patients with nasal obstruction are refractory to medical therapy [5]. In this study, it was demonstrated that the PAF receptor was located in eosinophils, macrophages, neutrophils, mast cells, lymphocytes, vascular endothelial cells, epithelial cells, and submucosal glands [5]. However, no PAFR gene or protein expression has been reported in the nasal tissues of patients suffering from chronic rhinosinusitis with nasal polyps (CRSwNP).

Similarly, to most cell receptors, the concentration of PAF plays an important role in regulating the level of PAFR activation. In fact, it has been reported that the nasal polyps (NP) from patients with aspirin/NSAID-exacerbated respiratory disease (N-ERD) contained relatively large amounts of PAF. Moreover, it has been demonstrated that PAF activity correlated with tissue eosinophilia, which is a characteristic of the nasal polyp tissue of patients suffering from N-ERD. These findings suggest that the PAF/PAFR system could be upregulated in the upper airways of N-ERD patients [6].

The concentration of PAF is determined, in part, via the balance between enzyme synthesis and degradation/inactivation. In fact, PAF has an acetyl group at the sn-2 position of its glycerol backbone, which is essential for its biological activity. The deacetylation reaction results in the formation of less active metabolites, e.g., Lyso-PAF [7]. Similarly to PAF, the existence of several molecular species of Lyso-PAF have been reported, such as C16 Lyso-PAF, C18 Lyso-PAF, and C18:1 Lyso-PAF [8]. Despite the fact that Lyso-PAF isoforms could play a role in the regulation of the PAF/PAFR system, no Lyso-PAF analysis has been reported in nasal mucosa (NM) tissues from healthy subjects and NP tissues from CRSwNP patients with/without asthma.

The effects of PAF and Lyso-PAF in upper airway inflammation have been investigated through different in vivo, in vitro, animal, and human experimental models. PAF elevates nasal symptoms in both healthy volunteers and allergic rhinitis patients. The instillation into both nasal cavities of PAF with increasing concentrations results in non-atopic healthy volunteers and allergic rhinitis patients out of pollen season, which results in increased nasal symptom scores in both groups, with no differences in the response to the PAF nasal challenge between non-atopic healthy volunteers and allergic rhinitis patients [9,10]. In line with these findings, inhaled PAF on guinea pig nasal mucosa evoked rhinitis-like symptoms [11], while a rat model of rhinosinusitis has been developed through intranasal application of PAF [12]. The mechanisms of PAF include the regulation of pro-inflammatory cells and mediators, such as increases in eosinophil cationic protein (ECP) concentration [13], as well as increments in the number of eosinophils [14] and neutrophils [15] in the nasal lavage. In fact, nasal challenges with PAF in atopic and non-atopic subjects resulted in eosinophilia in nasal lavage that was faster in atopic patients than in non-atopic subjects [15]. Apart from its direct effects, PAF increases the reactivity of the nasal mucosa to histamine and bradykinin. Intranasal PAF aerosol administration to normal human subjects induced an increased responsiveness to inhaled histamine and bradykinin [13]. However, there is limited information regarding the pro-inflammatory effects and mechanisms of PAF in the pathogenesis of CRSwNP.

Despite Lyso-PAF being a metabolite product of PAF, Lyso-PAF could also play a role in regulating the concentration of PAF. In human-cultured sinus mucosa from control subjects, Lyso-PAF was transformed into PAF, suggesting that the turnover of Lyso-PAF to PAF may play a role in evoking prolonged inflammation in target organs or tissues [16]. Using human paranasal sinus mucosa cultures, it has been demonstrated that after the addition of Lyso-PAF, PAF became detectable in the culture medium at 10 min, and reached a maximum concentration at 20 min. The PAF level then gradually declined to become undetectable at 60 min following the addition of Lyso-PAF. Thus, it seems that PAF is very unstable, having a half-life calculated to be 12.8 min. In contrast, since Lyso-PAF is known to be a stable metabolite, the results obtained from this study suggested that the turnover of Lyso-PAF to PAF may play a role in evoking prolonged inflammation in target organs or tissues.

In addition, the nasal challenge with Lyso-PAF in atopic and non-atopic subjects resulted in neutrophilia after 3 h after stimulation. Moreover, an increase in eosinophil counts was observed 3 h after Lyso-PAF stimulation in atopic subjects but not in non-atopic subjects [15]. On the contrary, it has been reported that in control subjects and allergic rhinitis patients, intranasal administrations of Lyso-PAF did not increase the reactivity of the nasal airway in response to histamine [13] nor induced nasal symptoms or nasal airway resistance [9]. However, the effects of Lyso-PAF in the upper airways of CRSwNP patients have not been reported.

In summary, despite the role of the PAF/PAFR system in the pathogenesis of allergic rhinitis appearing to be clearly demonstrated, the role of PAFR and Lyso-PAF isoforms in the mechanisms of action in CRSwNP remain to be elucidated. The objectives of this study were to assess the expression of PAFR and the concentration of Lyso-PAF isoforms in the NP of patients suffering from CRS with/without comorbidities (e.g., asthma and N-ERD) compared to the NM from healthy subjects.

## 2. Materials and Methods

### 2.1. Study Population and Design

Nasal mucosa specimens were obtained from 8 patients (6 men and 2 women), ranging from 27 to 52 years old (40.6 years ± 7.6), undergoing nasal corrective surgery for septal dysmorphia, turbinate hypertrophy, or both. A skin prick test was positive in 4 patients (50%). Nasal polyp specimens were obtained from 18 patients (12 men and 6 women), ranging from 22 to 76 years old (52.5 years ± 14.7), undergoing endoscopic sinus surgery (ESS) with nasal polypectomy. A skin prick test was positive in 5 patients (41.7%). Three different CRS with NP phenotypes were studied: (1) with no type 2 respiratory comorbidities (CRS NP; *n* = 6), (2) with aspirin/NSAID-tolerant asthma (ATA NP; *n* = 6), and (3) with aspirin/NSAID-exacerbated respiratory disease (N-ERD NP; *n* = 6). The clinical diagnosis of CRSwNP was based on the presence of sinonasal symptoms, the presence of nasal polyps via nasal endoscopy, and bilateral sinus opacification detection via computed tomography scans, following the European Position Paper on Rhinosinusitis and Nasal Polyps (EPOS) criteria. Asthma and N-ERD were clinically diagnosed by an allergologist through bronchial symptoms and diagnostic tools (e.g., spirometry and lysine acetylsalicylate provocation test). The same exclusion criteria were applied for control subjects and patients. Individuals receiving systemic corticosteroids, H1 antihistamine (including those with dual anti-PAF effect), antileukotriene, or biologic treatment 4 weeks prior to their ESS or having an upper or lower airway infection 2 weeks prior to their ESS were excluded from this study. All patients gave informed consent to participate in this study at the time of surgery. The tissues used in this study were obtained from the tissue collection BTIRCE-R100311-016 belonging to the IDIBAPS Tissue and Cell Bank. The Scientific and Ethics Committee of our institution approved the study project (HCB/2016/0635).

### 2.2. Tissue Handling

NM and NP tissue specimens were removed via surgery from ESS and immediately transported to the laboratory and processed according to the tissue protocol of specific analysis:

1. PAFR immunohistochemistry. Pieces of tissues (about 5 mm diameter) were immersed in 4% neutral-buffered formalin for 24 h and then dehydrated and cleared using increasing ethanol concentrations. Finally, they were embedded into immunohistochemistry grade paraffin and stored at room temperature until microtome sections were performed.
2. PAFR protein (Western blot) and mRNA (reverse transcription-quantitative PCR (RT-qPCR)). Pieces of tissues (about 5 mm diameter) were placed in cryogenic tubes and quickly frozen via immersion in liquid nitrogen. Frozen samples were stored at −80 °C until mRNA or protein isolation procedures were performed.
3. PAFR immunofluorescence. Pieces of tissues (up to 0.5 cm diameter) were embedded in OCT compound (Tissue-Tek; Sakura Finetek, Torrance, CA, USA) in cryomolds and quickly frozen by immersing it in liquid nitrogen. Samples were stored at −80 °C until frozen sectioning on a microtome cryostat.
4. Lyso-PAF analysis via gas–liquid chromatography–mass spectrometry (GLC/MS). Pieces of tissues (1 g of tissue) were placed in cryogenic tubes and quickly frozen via immersion in liquid nitrogen. Frozen samples were stored at −80 °C until Lyso-PAF analysis was performed.

### 2.3. Tissue Analyses

1. Protein location via immunohistochemistry. Paraffin-embedded tissue was cut in sections of 5–10 µm in a microtome and mounted on poly-L-lysine-coated glass slides. A protocol for the deparaffinization of paraffin-embedded sections was performed and, after permeabilization with sodium citrate (0.01 M; pH 6), the staining protocol was started. Samples were blocked with 4% hydrogen peroxide to reduce background staining before reducing nonspecific binding with 1:10 goat serum in PBS (Sigma-Aldrich Co., St. Louis, MO, USA) for 60 min at room temperature. Antibodies against PAFR (rabbit polyclonal anti-human PAFR, Novusbio NBP1-90346) at a dilution of 1:400 were incubated overnight at 4 °C. Immunoreactivity of PAFR was detected with the EnVision+ System-HRP using an Olympus microscope. Controls using samples treated with either primary or secondary antibodies alone were performed with no specific immunoreactivity.
2. Protein expression via Western blot analysis. Total proteins were isolated from frozen tissues using RIPA lysis buffer. Tissue samples were placed in tubes containing ice-cold RIPA buffer (50 mM Tris-HCl (pH 7.4), 150 mM NaCl, 0.1% SDS, 1% Nonidet P-40 (IGEPAL), 1 mg/mL leupeptin, 1 mg/mL aprotinin, 0.1 mM Na_3_VO_4_, 1 mM NaF, 1 mM dithiothreitol, 0.5 mg/mL Pefabloc, and 5 mg/mL sodium deoxycholate (Sigma)). Samples were kept on ice and sonicated twice for 15 s in a sonifier (Branson, Danbury, Conn) before centrifugation at 12,000× *g* for 10 min at 4 °C. Twenty micrograms of protein were denatured in a thermocycler (70 °C for 10 min) in loading buffer (NuPAGE LDS sample buffer), loaded onto 7% SDS–polyacrylamide gels, and electrophoresed at 125 V for 90 min in a Novex XCell II Mini-Cell (Invitro-gen). Finally, proteins were transferred to nitrocellulose membranes, and nonspecific binding sites were blocked with blocking buffer (5% non-fat dry milk and 0.1% Tween-20 in 10 nM PBS) for 1 h at room temperature in an orbital shaker. The membranes were then incubated in blocking buffer overnight at 4 °C with primary antibodies against PAFR (rabbit polyclonal anti-PAFR antibody, Abcam, ab104162) at a 1:500 dilution or incubated for 2 h at room temperature with primary antibodies against β-actin (mouse monoclonal to β-actin HRP conjugated, abcam, ab49900) at a dilution of 1:10,000. After this period, the blots were washed in 0.05% Tween-20 in 10 nM PBS. In the case of membranes incubated with anti-PAFR antibody, a second incubation with a goat anti-rabbit IgG-HRP (Santa Cruz Biotechnology, Inc. Dallas, TX, USA, dilution 1:3000) at room temperature for 2 h was performed. After repeated washes, the blots were incubated with an enhanced chemiluminescent Immobilon^®^ Forte Western HRP substrate (Cat No WBLUF0100, Millipore, Burlington, MA, USA), and light emissions were detected using a CCD Camera System LAS 4000 (Fujifilm, Tokyo, Japan). Band intensities were quantified with ImageQuantTL Software v8.2.0. Protein signals were normalized to those of β-actin.
3. Protein location via Immunofluorescence. Frozen tissues embedded in OCT were cut in sections of 5–10 µm in a cryostat and mounted on gelatin-coated histological slides. Sections were air dried for 30 min at room temperature and fixed for 8 min by adding 50 µL of ice-cold fixation buffer. Tissues were permeabilized with 0.2% Triton X-100 (Sigma-Aldrich Co.) in PBS (Sigma-Aldrich Co.), pH 7.4, at room temperature, and then blocked with 6% goat serum (Sigma-Aldrich Co.) for 60 min to prevent non-specific binding. Primary antibodies (anti-PRG2 antibody-BMK13, Abcam, ab14462) at a dilution of 1:100 and rabbit polyclonal anti-human PAFR (Novusbio, NBP1-90346) at a dilution of 1:300 were incubated overnight at 4 °C. Negative controls were performed by omitting the primary antibody and using a negative control serum of the same isotype. Alexa Fluor^®^ 488-conjugated AffiniPure donkey anti-rabbit IgG (H + L, green channel, cat number 149712, Jackson ImmunoResearch, West Grove, PA, USA) at a dilution of 1:300 and Alexa Fluor 555-conjugated Donkey anti-Mouse IgG (H + L, red channel) (Invitrogen, A-31570) at a dilution of 1:500 were used as the secondary antibodies. The samples were counterstained with DAPI to identify cell nuclei (blue channel), and the slides were cover-slipped with ProLong Gold Antifade reagent (Invitrogen, Waltham, MA, USA).
4. Lyso-PAF via LC-MS/MS analysis. A LC-MS/MS method for the determination of Lyso-PAF C16 (Cayman Chemical, Ann Arbor, MI, USA, 60906), Lyso-PAF C18 (Cayman Chemical, 60916), and Lyso-PAF C18:1 (Avanti Polar Lipids, Birmingham, AL, USA, 878126C) in the nasal mucosa and nasal polyp samples was developed in the range of 0.2–20 ng/mL for the three components. Samples (approximately 0.5 g of tissue) were collected in 1 mL of methanol (Scharlab, Quezon City, Philippines, ME0306) and, after sample homogenization, 100 µL were processed with a liquid–liquid extraction method with chloroform (Scharlab, CL0207). The curves and QCs for the quantification of Lyso-PAF C16, C18, and C18:1 were prepared in H_2_O in the range of 0.2–20 ng/mL due to the lack of selectivity and the presence of interferents in matrices. The Lyso-PAF compound C18-d4 (Cayman Chemical, 10010228) was used as an internal standard. The HPLC separation of Lyso-PAF C16, C18, and C18:1 was carried out with a gradient and reverse phase chromatography with an XSelect CSH C18 column (130 Å; 3.5 µm; 2.1 × 100 mm) (Waters, 186005256) and 5 mM ammonium acetate (Supelco, Washington, DC, USA, 73594)/methanol (LC-MS, Scharlab ME0326) mobile phases, with retention times of 5.7 min, 6.3 min, and 8.3 min for Lyso-PAF C16, C18:1, and C18, respectively. MS/MS detection was performed using a Sciex 6500+ triple quadrupole mass spectrometer (ionization in positive mode) to monitor the most abundant MRM fragments *m*/*z* 482→*m*/*z* 104, *m*/*z* 508→*m*/*z* 104, and *m*/*z* 510→*m*/*z* 104 for Lyso-PAF C16, C18:1, and C18, respectively. The LC-MS/MS method provided sufficient sensitivity, precision, and accuracy for the determination of the compounds Lyso-PAF C16, C18:1, and C18 in samples of the NM and NP.
5. mRNA expression via RT-qPCR analysis. Total RNA was isolated from frozen tissues using TRIzol reagent (Life Technologies, Carlsbad, CA, USA), according to the manufacturer’s protocol. RNA was reverse transcribed to cDNA using the High-Capacity cDNA Reverse Transcription kit, according to the manufacturer’s instructions (Thermo Fisher, Waltham, MA, USA). PAFR mRNA expression was analyzed via real-time qPCR using TaqMan gene expression assays (Life Technologies) in the RT 7900 Real-Time PCR system (Thermo Fisher) with QuantStudio^TM^ software v1.3. The TaqMan gene expression assays were glyceraldehyde-3-phosphate dehydrogenase (GAPDH, Hs02758991_g1) and platelet-activating factor receptor (PAFR, Hs00265399_s1). The analysis of results was performed using the 2^(−DCt) method.
6. Statistical data analysis: Data were given as the median and interquartile range. Whiskers represent the minimum and maximum values. Multiple comparisons were analyzed using the one-way analysis of variance statistical test with Tukey/Newman-Keuls post-hoc analysis or with Kruskal–Wallis with Dunn’s post-hoc analysis, as appropriate. Comparisons between two groups were made with the *t*-test or the Mann–Whitney U test, as appropriate. Statistical analyses were performed with GraphPad Prism software (GraphPad Prism Software v8.4.0, La Jolla, CA, USA). Statistical significance was set at *p* < 0.05.

## 3. Results

### 3.1. Analysis of PAFR Protein and Gene Expression

**Figure 1:**
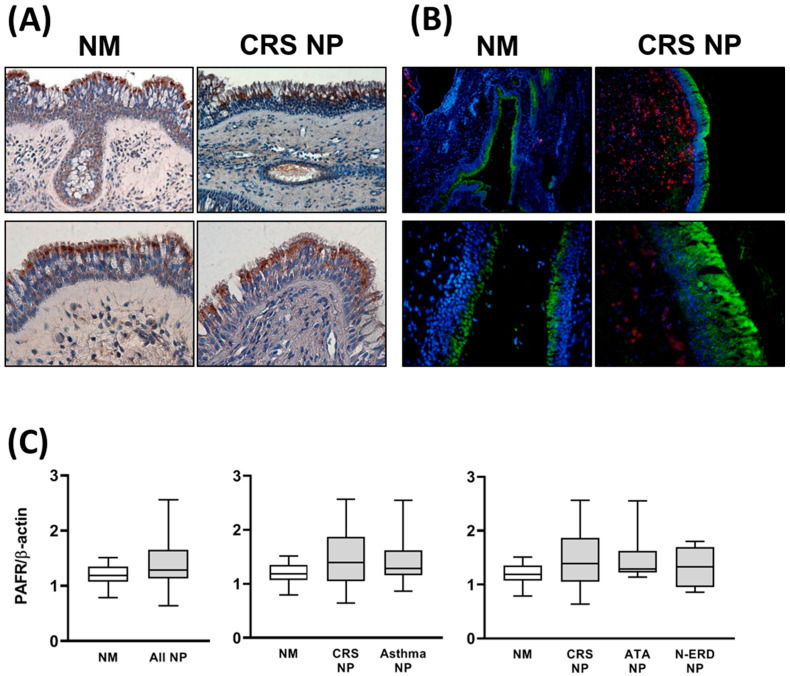
PAF receptor (PAFR) protein expression in nasal mucosa (NM) and nasal polyp (NP) tissues. PAFR protein immunohistochemistry (brown) (**A**) and immunofluorescence (green) (**B**) images in nasal mucosa (NM) from control subjects and nasal polyps (NP) from chronic rhinosinusitis (CRS) patients without asthma. Original magnification: 200×–400×. (**C**) PAFR protein expression was analyzed via Western blot in the NM from control subjects (*n* = 8), NP from CRS patients without asthma (CRS NP; *n* = 6), NP from CRS patients with asthma (asthma NP; *n* = 12), NP from CRS patients with NSAID-tolerant asthma (ATA NP; *n* = 6), and NP from CRS patients with NSAID-exacerbated respiratory disease (N-ERD NP; *n* = 6). No differences were found between groups. The unpaired *t*-test was used for statistical comparisons.

The immunolocalization (via immunohistochemistry and immunofluorescence) of the PAFR protein showed positive immunoreactivity in epithelial cells and submucosal glands in both NM and NP tissues (Figure 1A,B). The immunolocalization in ATA NP and N-ERD NP was similar, despite lower immunoreactivity in the epithelial area due to the epithelial loss present in both tissues.

**Figure 2:**
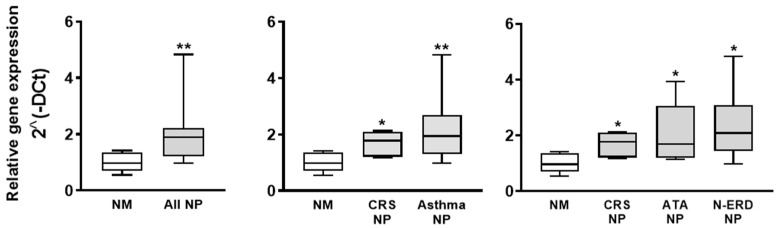
PAF receptor (PAFR) mRNA expression in nasal mucosa (NM) and nasal polyp (NP) tissues. PAFR gene expression analyzed via RT-qPCR in NM control subjects (*n* = 8), NP from chronic rhinosinusitis (CRS) patients without asthma (CRS NP; *n* = 6), NP from CRS patients with asthma (asthma NP; *n* = 12), NP from CRS patients with NSAID-tolerant asthma (ATA NP; *n* = 6), and NP from CRS patients with NSAID-exacerbated respiratory disease (N-ERD NP; *n* = 6). GAPDH was used as a house-keeping gene. The analysis was performed using the 2^(-DCt) method. The unpaired *t*-test was used for statistical comparisons. * *p* < 0.05 and ** *p* < 0.01 compared to NM.

The analysis via Western blot confirmed the presence of PAFR protein in all the tissues studied. No differences in PAFR protein expression were found when comparing NM and NP tissues (Figure 1C). The analysis of PAFR gene expression demonstrated that compared to NM tissues, mRNA expression was higher in all NP tissues. No differences in PAFR mRNA expression were found when comparing the different NP phenotypes (Figure 2).

### 3.2. Lyso-PAF Analysis in NM and NP Tissues

**Figure 3:**
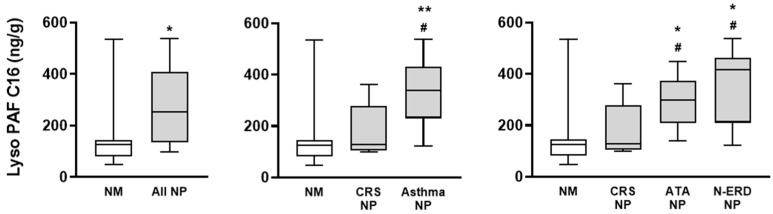
Lyso-PAF C16 concentration in nasal mucosa (NM) and nasal polyps (NP) tissues. Lyso-PAF C16 concentration was analyzed via gas–liquid chromatography–mass spectrometry in the NM from control subjects (NM; *n* = 8), NP from chronic rhinosinusitis (CRS) patients without asthma (CRS NP; *n* = 6), NP from CRS patients with asthma (asthma NP; *n* = 12), NP from CRS patients with NSAID-tolerant asthma (ATA NP; *n* = 6), and NP from CRS patients with NSAID-exacerbated respiratory disease (N-ERD NP; *n* = 6). The unpaired *t*-test was used for statistical comparisons. * *p* < 0.05 and ** *p* < 0.01 compared to NM; # *p* < 0.05 compared to CRS NP.

**Figure 4:**
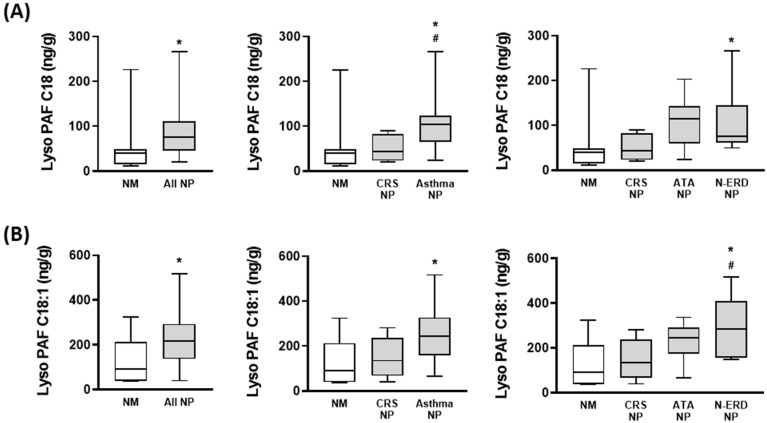
Lyso-PAF C18 and C18:1 concentration in nasal mucosa (NM) and nasal polyp (NP) tissues. Lyso-PAF C18 (**A**) and Lyso-PAF C18:1 (**B**) concentrations were analyzed via gas–liquid chromatography–mass spectrometry in the NM from control subjects (NM; *n* = 8), NP from chronic rhinosinusitis (CRS) patients without asthma (CRS NP; *n* = 6), NP from CRS patients with asthma (asthma NP; *n* = 12), NP from CRS patients with NSAID-tolerant asthma (ATA NP; *n* = 6), and NP from CRS patients with NSAID-exacerbated respiratory disease (N-ERD NP; *n* = 6). The unpaired *t*-test was used for statistical comparisons. * *p* < 0.05 compared to NM; # *p* < 0.05 compared to CRS NP.

The analysis of Lyso-PAF isoforms demonstrated that compared to the NM, the concentrations of Lyso-PAF C16, Lyso-PAF C18, and Lyso-PAF C18:1 were higher in the NP than in healthy nasal mucosa tissues (Figure 3 and Figure 4). When comparing different NP phenotypes with comorbidities, asthma NP, ATA NP, and N-ERD NP showed significantly higher Lyso-PAF C16 concentrations than CRS NP without asthma (Figure 3). Similarly, CRS NP with asthma showed a higher Lyso-PAF C18 concentration (Figure 4A), and N-ERD NP showed a higher Lyso-PAF C18:1 concentration (Figure 4B) than CRS NP without asthma.

## 4. Discussion

The main findings of our study are: (1) PAFR gene/protein expression and Lyso-PAF isoforms were present in both the NM from control subjects and in the NP from patients suffering from CRS; and (2) compared to healthy NM tissues, higher PAFR gene expression levels and higher concentrations of Lyso-PAF isoforms were found in inflamed NP tissues, predominantly in N-ERD patients.

Our study demonstrated the PAFR gene and protein expression in NM samples from control subjects and NP samples from CRS patients. To our knowledge, this is the first study showing the presence of PAFR in NM tissues obtained under non-inflammatory conditions. Shirasaki et al. reported that the PAFR protein and mRNA are present in the NM from patients with nasal obstruction refractory to medical therapy; however, no control subjects were included in the study [5]. Our findings suggest that no inflammatory conditions are required for PAFR gene expression in the NM.

We have also demonstrated, for the first time, that the PAFR gene and protein are expressed in NP tissues from CRS patients. Compared to the control NM, PAFR mRNA, but not protein, was upregulated in NP tissues. However, in keeping with findings in the NM, immunochemistry and immunofluorescence studies demonstrated PAFR protein immunolocalization in the epithelial cells and submucosal glands of the NP from patients with/without asthma. With the methodology used in our study, we could not discriminate whether PAFR mRNA upregulation found in NP tissues was related to inflammatory cell infiltration, upregulation of expression in epithelial cells and glands, or both mechanisms. Our results suggest that the PAF/PAFR system could play a pathophysiological role in CRSwNP, although no differences were found when comparing the different NP phenotypes. In addition, our findings indicate that comorbidities, such as asthma and N-ERD, do not significantly modify the level of PAFR mRNA expression in NP tissues.

In our study, we have also demonstrated for the first time that Lyso-PAF isoforms are present in both the NM of control subjects and NP of CRS patients. Moreover, NP from N-ERD patients showed an upregulated Lyso-PAF isoform concentration compared to NP from non-asthmatic patients, suggesting an association between upper airway inflammation and disease severity with Lyso-PAF concentrations. These findings are in accordance with the results reported by Furukawa et al., who showed that the PAF concentration in human NP was higher in those with severe eosinophil infiltration [6].

In fact, circulating eosinophils in CRSwNP patients occur before their migration to the NP, reflecting the systemic nature of CRSwNP compared to healthy controls [17]. Moreover, increased Lyso-PAF isoform concentrations could play a role in the pathogenesis of CRSwNP through mechanisms demonstrated in the human NM, such as increasing tissue neutrophilia and eosinophilia [15] and its metabolic transformation to PAF [16].

In summary, this study demonstrates the upregulation of PAFR gene expression as well as the increased concentration of Lyso-PAF isoforms in NP tissues. However, due to the small sample size included in our study, further research is needed to confirm our findings and to potentially develop new anti-PAF therapies.

## 5. Conclusions

Both PAFR mRNA and protein were found expressed in NM and NP tissues. Moreover, NP tissues showed an upregulation of PAFR mRNA expression as well as Lyso-PAF isoforms compared to healthy NM tissues, suggesting that the PAF/PAFR system could play a pathophysiological role in the pathogenesis of CRSwNP, opening the possibility of developing anti-PAF drugs to treat patients with CRSwNP.

## Data Availability

The data presented in this study are available on request from the corresponding author. The data are not publicly available due to privacy restrictions.
